# Secondary Metabolite Profiling, Antioxidant, Antidiabetic and Neuroprotective Activity of *Cestrum nocturnum* (Night Scented-Jasmine): Use of In Vitro and In Silico Approach in Determining the Potential Bioactive Compound

**DOI:** 10.3390/plants12061206

**Published:** 2023-03-07

**Authors:** Saheem Ahmad, Mohammed Alrouji, Sharif Alhajlah, Othman Alomeir, Ramendra Pati Pandey, Mohammad Saquib Ashraf, Shafeeque Ahmad, Saif Khan

**Affiliations:** 1 Department of Medical Laboratory Sciences, College of Applied Medical Sciences, University of Hail, Hail 2440, Saudi Arabia; 2 Department of Clinical Laboratory Sciences, College of Applied Medical Sciences, Shaqra University, Shaqra 11961, Saudi Arabia; 3 Department of Pharmacy Practice, College of Pharmacy, Shaqra University, Shaqra 11961, Saudi Arabia; 4 Department of Biotechnology, SRM University Delhi-NCR, Sonepat 131 029, India; 5 Department of Clinical Laboratory Sciences, College of Applied Medical Sciences, Riyadh ELM University, Riyadh 12734, Saudi Arabia; 6 Department of Biochemistry, Noida International Institute of Medical Sciences, Noida International University, Gautam Budh Nagar 203 201, India; 7 Department of Basic Dental and Medical Sciences, College of Dentistry, University of Hail, Hail 2440, Saudi Arabia

**Keywords:** diabetes, α-amylase, acetylcholinesterase, Alzheimer, secondary metabolites, night blooming jasmine

## Abstract

This study aims to describe the therapeutic potential of *C. nocturnum* leaf extracts against diabetes and neurological disorders via the targeting of α-amylase and acetylcholinesterase (AChE) activities, followed by computational molecular docking studies to establish a strong rationale behind the α-amylase and AChE inhibitory potential of *C. nocturnum* leaves-derived secondary metabolites. In our study, the antioxidant activity of the sequentially extracted *C. nocturnum* leaves extract was also investigated, in which the methanolic fraction exhibited the strongest antioxidant potential against DPPH (IC_50_ 39.12 ± 0.53 µg/mL) and ABTS (IC_50_ 20.94 ± 0.82 µg/mL) radicals. This extract strongly inhibited the α-amylase (IC_50_188.77 ± 1.67 µg/mL) and AChE (IC_50_ 239.44 ± 0.93 µg/mL) in a non-competitive and competitive manner, respectively. Furthermore, in silico analysis of compounds identified in the methanolic extract of the leaves of *C. nocturnum* using GC-MS revealed high-affinity binding of these compounds with the catalytic sites of α-amylase and AChE, with binding energy ranging from −3.10 to −6.23 kcal/mol and from −3.32 to −8.76 kcal/mol, respectively. Conclusively, the antioxidant, antidiabetic, and anti-Alzheimer activity of this extract might be driven by the synergistic effect of these bioactive phytoconstituents.

## 1. Introduction

Oxidative stress induced by reactive oxygen species (ROS) is deleterious to proteins, lipids, cell membranes, and DNA, and contributes to the development of several chronic and degenerative disorders [1]. An imbalance between oxidative stress and the antioxidant defense system causes cellular dysfunction, resulting in the development of many chronic diseases, including diabetes mellitus (DM) and neurological disorders [2,3]. DM is a metabolic disorder characterized by impaired carbohydrate metabolism resulting in elevated fasting and postprandial blood sugar levels. During persistent hyperglycemia, glucose can react with proteins nonenzymatically through the process of glycation [4,5]. Glycation of proteins and formation of advanced glycation end products are involved in the pathogenesis of several diabetic complications, including neurological dysfunction [6,7]. The prevalence of diabetes is increasing globally—approximately 537 million adults lived with diabetes in 2021 and the disease accounted for more than 6.0 million fatalities, half of which were in cases that were still undiagnosed. These numbers are predicted to increase to ~645 and ~785 million by 2030 and 2045, respectively [8].

Alzheimer’s disease (AD) is the most common form of dementia, with memory loss, language inability, cognitive dysfunction, visuospatial skill deficiency, and difficulty in judgement being the most common symptoms [9,10]. Moreover, abnormal accumulation of β-amyloid in the synaptic cleft of the neurons and of tau-neurofibrillary tangles plaques inside it disrupt the neuronal function [11]. Acetylcholine (ACh) is a chemical released at the neuromuscular junction that acts as a neurotransmitter (chemical message) allowing interneuronal communication. In the synaptic cleft, free ACh is synthesized by acetylcholinesterase (AChE) and it is ensured that no excess ACh is present for continuous activation of receptors [11]. Although the underlying cause of AD remains unclear, the pathogenesis is firmly associated with cholinergic transmission dysfunction. The inhibition of AChE is a widely accepted therapeutic strategy for symptomatic treatment of AD [12].

The incidence of both DM and AD is increasing. Moreover, diabetic patients have a five-fold higher risk of developing AD than nondiabetic individuals [3,13,14,15]. Diabetes patients also show reduced baseline cognitive abilities, such as those related to memory, learning, and judgment [13]. The relationship of hyperglycemia and insulin signaling anomalies with AD has been reported to be strong; because of which, AD is often considered as a metabolic brain disease [10,16,17]. DM and AD share a common pathophysiology, involving oxidative stress, inflammation [18], high cholesterol levels, neuronal degeneration, β-amyloid accumulation [19], phosphorylation of tau protein, and glycogen kinase-3 synthesis [20].

Antidiabetic drugs that reduce insulin resistance in the brain could prevent AD or dementia [15]. However, despite their impactful therapeutic response against DM and AD, such drugs fail to reverse the complications and are associated with prominent side effects [21]. Thus, alternative natural sources are being explored for therapeutic compounds effective against both DM and AD that would less likely be associated with complications. Strategies aimed at reducing oxidative stress and delaying the absorption of glucose and ACh synthesis via inhibition of α-amylase and AChE have the potential for effective management of DM and AD.

In this context, in the present study, we screened the antidiabetic and anti-Alzheimer’s potential of *Cestrum nocturnum*, a solanaceous shrub widely found in tropical and subtropical countries, including Australia, China, India, and America [22]. The leaves are simple, narrow lanceolate, smooth, and glossy, with an entire margin. *C. nocturnum* has garnered the attention of researchers in view of its antioxidative [23], antimicrobial [24], antifungal [22,24], anti-inflammatory [25], and hepatoprotective properties [26]. The antidiabetic and antihyperlipidemic activities of *C. nocturnum* have been reported in rodents [27,28]. In addition, several bioactive phytoconstituents such as flavonoids, glycosides, tannins, coumarins, anthocyanins, sapogenins, and sterols have been also identified, which have numerous biological activities such as antibacterial, antifungal activities [22]. In this study, for the first time, we evaluated the efficacy of extracts of the leaves of *C. nocturnum* as potent dual inhibitors of α-amylase and AChE. In addition, molecular docking studies of secondary metabolites in the methanolic (MeOH) extract of leaves of *C. nocturnum* identified using gas chromatography-mass spectrometry (GC-MS) analysis were performed to obtain mechanistic insights into their inhibitory activities.

## 2. Results

### 2.1. Phytochemical Screening and Total Phenolic Content in Extracts of the Leaves of C. nocturnum

*C. nocturnum* leaves were sequentially extracted in *n*-Hexane, dichloromethane (DCM), ethyl acetate (EtOAc), methanol (MeOH), and water. The percent yield of extraction is shown in Table 1. Phytochemical screening revealed significant amounts of bioactive compounds, including flavonoids and polyphenols (Table 2) with free radical quenching ability, in the MeOH extract. The reductones serve as antioxidants by donating a hydrogen to the free radical, often corresponding with the reducing capacity of compounds, which may be a significant signal of its antioxidant potential [29,30,31].

### 2.2. α,α-Diphenyl-β-picrylhydrazyl (DPPH) Assay

**Figure 1:**
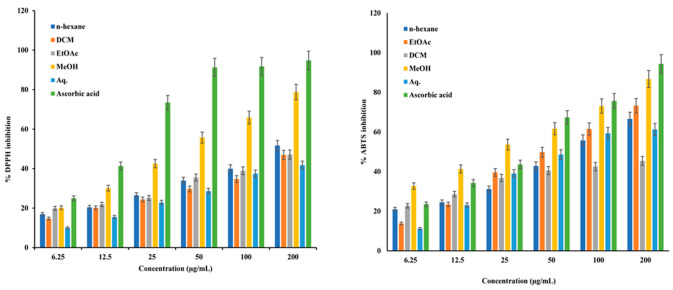
Free radical scavenging and antioxidant potential of different extracts of the leaves of *C. nocturnum* measured using the DPPH and ABTS assays. Bar graph represents the % inhibition of radicals. The values are represented as mean ± SD of data from triplicate assays.

DPPH is a relatively stable radical that is widely used to evaluate the quenching ability of antioxidants from natural sources such as fruit and plant extracts. The DPPH scavenging ability of different extracts of the leaves of *C. nocturnum*, at various concentrations, is presented as % inhibition in Figure 1. The MeOH extract, with an IC_50_ value of 39.12 ± 0.53 µg/mL, was found to be the most potent in neutralizing the DPPH radical. The IC_50_ of the reference standard, ascorbic acid, was 15.12 ± 0.65 µg/mL (Table 3).

### 2.3. ABTS Radical Scavenging Assay

The ABTS radical cation scavenging assay is widely used to evaluate the antioxidant potential of plant and fruit extracts and purified compounds. All the extract of the leaves of *C. nocturnum* neutralized the ABTS radical in a dose-dependent manner via electron donation to the radical (Figure 1). The inhibition of the ABTS radical was highest for the MeOH fraction (IC_50_ 20.94 ± 0.82 µg/mL) and that for the standard, ascorbic acid, was 94.33% (IC_50_ 22.76 ± 0.43 µg/mL) (Table 3). The percent inhibition by each fraction has been shown in the Figure 1.

### 2.4. Ferric Reducing Antioxidant Power (FRAP)

**Figure 2:**
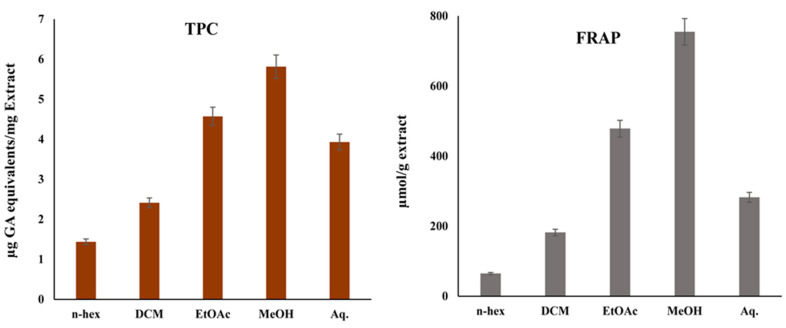
Ferric reducing antioxidant power (FRAP) and total phenolic content (TPC) of *C. nocturnum* leaf extracts. The values are represented as mean ± SD of data from three parallel assays.

The FRAP assay was used to evaluate the ferric-reducing potential of distinct extracts of the leaves of *C. nocturnum*. The outcomes demonstrated that MeOH has considerably higher FRAP values, 478.50 ± 4.56 µmol Fe (II)/g, compared to other extracts (Figure 2).

### 2.5. Total Phenolic Content

The TPC was the highest in the MeOH extract (5.81 ± 0.2 µg gallic acid (GA) equivalents/mg extract) and lowest in the *n*-Hexane extract (1.43 ± 0.23 µg GA equivalents)/mg extract) (Figure 2).

### 2.6. Evaluation of α-Amylase Inhibition and Kinetics Studies to Explore the Mode of Action of the Extract

**Figure 3:**
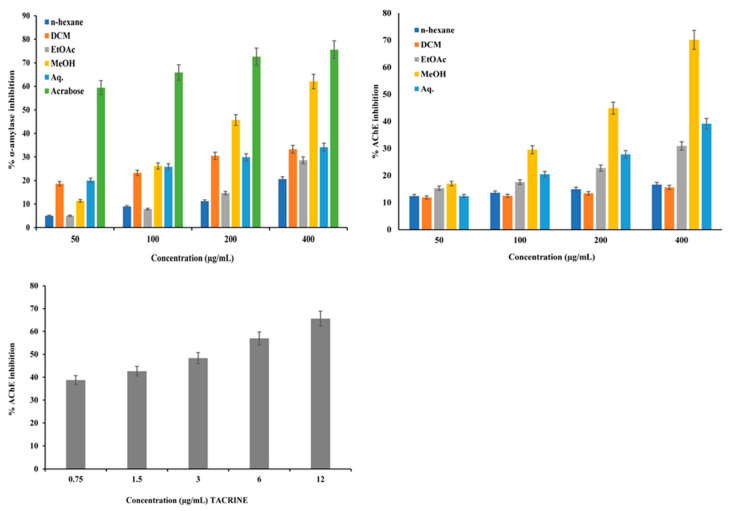
In vitro inhibition of α-amylase and acetylcholine esterase (AChE) activities by *C. nocturnum* leaf extracts. The MeOH extract was the most potent in inhibiting α-amylase. Bar graph represents the % inhibition of α-amylase and AChE. The values are represented as mean ± SD of data from triplicate assay.

**Figure 4:**
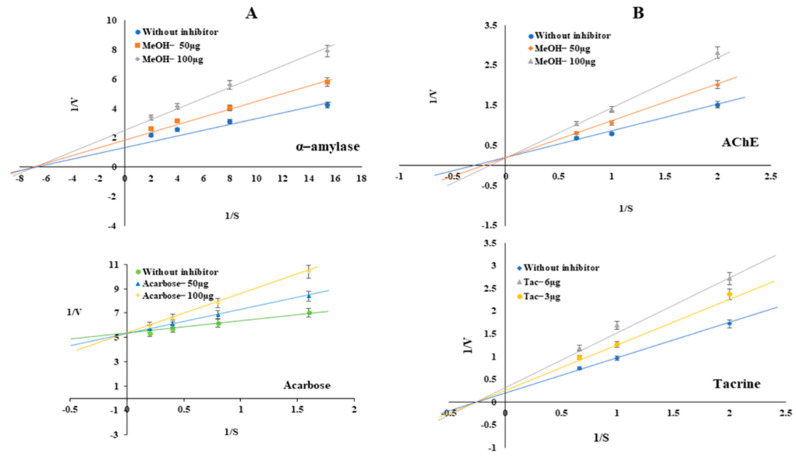
Kinetics of α-amylase and AChE inhibition by the MeOH extract of leaves of *C. nocturnum* and standard drugs (acarbose and tacrine). Kinetics of inhibition of α-amylase (**A**) and AChE (**B**). Different concentrations of substrate and inhibitors were used to evaluate the mode of inhibition. The Lineweaver–Burk plot was plotted using the 1/S vs. 1/V values. The plots show that the MeOH extract inhibits the α-amylase and AChE activities in a noncompetitive and competitive manner, respectively.

To investigate the antidiabetic activity, the α-amylase inhibitory potential of different extracts was evaluated. The MeOH extract effectively inhibited α-amylase in a dose-dependent manner and had the lowest IC_50_ value of 188.77 ± 1.67 µg/mL compared with those of the other extracts (Figure 3, Table 3). The standard drug, acarbose, showed 75.58% inhibition of α-amylase (IC_50_ 41.54 ± 0.54 µg/mL) (Figure 3, Table 3). Furthermore, kinetics studies revealed noncompetitive inhibition of α-amylase by the MeOH extract unlike the competitive inhibition by acarbose (Figure 4).

### 2.7. Evaluation of Acetylcholinesterase Inhibition and Kinetics Studies to Explore the Mode of Action of the Extract

The AChE enzyme activity was evaluated using a colorimetric method in which a yellow-colored 5-thionitrobenzoate anion, with an absorption maximum at 412 nm, is produced when thiocholine reacts with 5,5-dithio-bis-(2-nitrobenzoic acid) (DTNB). Amongst the five *C. nocturnum* leaf extracts, the MeOH extract exhibited the highest AChE inhibitory activity in a dose-dependent manner, with an IC_50_ of 239.44 ± 0.93 µg/mL (Figure 3, Table 3). The standard drug, tacrine, showed the lowest IC_50_ of 4.03 ± 0.47 µg/mL (Figure 3, Table 3). A kinetics study was performed to determine the mode of inhibition by tacrine and the MeOH extract. As is evident from the Lineweaver–Burk double reciprocal plot of 1/V vs. 1/[S] (Figure 4), the MeOH fraction showed a competitive inhibition, whereas Tac exhibited a noncompetitive inhibition, indicating that it binds to the allosteric site of the enzyme (Figure 4).

### 2.8. GC-MS Analysis

The MeOH extract, which showed the highest antioxidant potential and significantly inhibited α-amylase and AChE, was subjected to the GC-MS analysis to determine its phytoconstituents. A total of 23 compounds were identified by comparing the GC-MS spectra against a reference (NIST) library (Table 4). The three major compounds in the MeOh extract were found to be 2,4-Di-tert-butylphenol (20.05%), Precocene I (18.76%), and Hexaglycerine (14.46%), whereas Methyl 3-(3,5-ditert-butyl-4-hydroxyphenyl) propanoate (6.73%), DL-arabinose (4.11%), and Eicosanebioic acid (3.70%) were present in lesser amounts. Some other compounds were also found to be present in minute quantities (0.10–2.44% peak area) (Table 4). The chromatograms of the GC-MS identified compounds has been provided in Supplementary material (Supplementary File S1).

### 2.9. ADME Profiling of Compounds Identified via GC-MS Analysis

In this technical era, various computational strategies for the assessment of absorption, distribution, metabolism, excretion, and toxicology (ADMET) have been developed to reduce the time, money, and manpower in the field of drug discovery. In this context, we have performed the ADME analysis via an online web server, SwissADME, to unravel the physiochemical properties and pharmacokinetic profile of compounds identified via GC-MS analysis. In the BOILED-Egg analysis, 3 compounds were in the white region, predicted to have a higher intestinal absorption, whereas 11 compounds were in the yolk region, which were predicted to have a higher potential for penetration across the blood–brain barrier. Four compounds were outside the acceptable range and five compounds did not come under the definition of a “BOILED-Egg.” In the analysis of drug-like properties, Lipinski’s rule of five, bioactivity profile, and ADMET properties of the selected compounds were determined using the AI-based software. The five criteria in the Lipinski’s rule, viz. molecular weight <500 Da, H-bond donors (HBD) <5, H-bond acceptors (HBA) < 10, and Log P (octanol–water partition coefficient) <5, were evaluated for each of the compounds. In the drug-likeness analysis, compounds 1, 2, 8, 11, 16, 18, 21, and 23 violated the one rule (MlogP < 4.15), whereas compounds 15, 17, 20, and 22 violated two rules (MlogP < 4.15 and MW < 500) of Lipinski (Table 5).

### 2.10. Toxicity Assessment of the Selected Compounds

The compounds that resided in the BIOLED-Egg region were subjected to the toxicity analysis via ProTox-II, an online web server tool that predicts the toxicity class, LD_50_, and distinct toxicity parameters, such as hepatotoxicity, carcinogenicity, immunogenicity, mutagenicity, and cytotoxicity. Four compounds (dibutyl phthalate, phthalic acid di-isobutyl ester, *p*-chloromethoxybenzene, and precocene I) were predicted to be carcinogenic. Precocene I was also predicted to be immunogenic (Table 6). These compounds were, therefore, eliminated from further docking analysis.

### 2.11. Selected Compounds Actively Occupied the Active Pocket of α-Amylase and AChE

**Figure 5:**
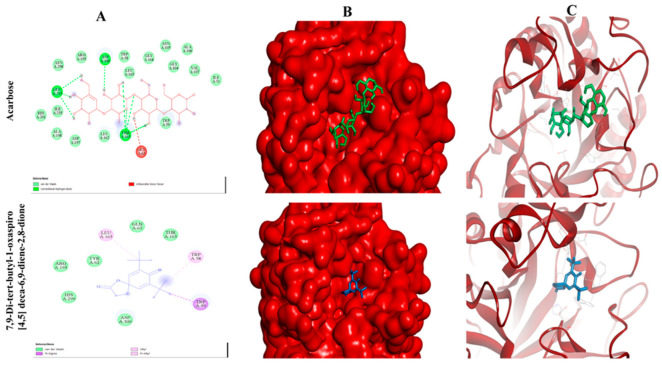
Molecular binding patterns within the active pocket of α-amylase crystal structure. Panel (**A**): 2-D ball and stick model representation. Panel (**B**): The binding pattern representation on protein surface. Panel (**C**): Interaction of inhibitor with α-amylase surrounded by α-helix and β-sheet conformations.

**Figure 6:**
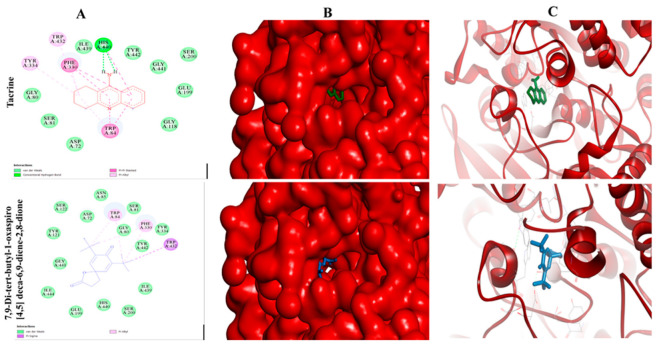
Molecular binding patterns within the active pocket of AChE crystal structure. Panel (**A**): 2-D ball and stick model representation. Panel (**B**): The binding pattern representation on protein surface. Panel (**C**): Interaction of inhibitor with AChE surrounded by α-helix and β-sheet conformations.

In this attempt, we found that all the selected compounds actively occupied the catalytic site of both the α-amylase and AChE crystal structure, with binding energy values ranging from −3.10 to −6.23 kcal/mol (Table 7) and −3.32 to −8.76 kcal/mol (Table 8), respectively. The grid box dimensions for α-amylase and AChE were 60 × 60 × 60 points (x, y, and z), with a grid spacing of 0.563 Å and 0.525 Å, respectively. The grid center at dimensions of x, y, and z for α-amylase and AChE were 14.56, 86.21, 153.11, and 3.4, 67.1, and 67.0, respectively. The docked complexes showed that a compound, namely 7,9-Di-tert-butyl-1-oxaspiro [4,5] deca-6,9-diene-2,8-dione, was found to be the most potent inhibitor of α-amylase and AChE, with binding affinity of -6.23 and -8.76 kcal/mol, respectively, which is better than their respective standard and substrate, while other compounds also showed significant binding affinity (Table 7 and Table 8). The docked complex of α-amylase and AChE with 7,9-Di-tert-butyl-1-oxaspiro [4,5] deca-6,9-diene-2,8-dione was found to be surrounded by 9 amino acid residues (Leu165, Gln63, Thr163, Trp58, Trp59, Asp300, His299, Arg195, Tyr62) (Figure 5) and 17 amino acids residues (Ser122, Asp72, Asn85, Trp84, Ser81, Gly80, Phe330, Tyr334, Tyr442, Trp432, Ile439, Ser200, His440, Glu199, Ile444, Gly441, Tyr121), respectively (Figure 6).

## 3. Discussion

Oxidative stress induced by ROS damages proteins, lipids, and DNA and is one of the major causes of several chronic diseases, such as DM [29,32]. The prevalence of DM is rising globally and this trend is predicted to continue in the coming decades [8]. Oxidative stress and DM are independent risk factors for several complications, including cardiovascular diseases, diabetic encephalopathy, and AD [2,32,33,34,35]. Antioxidants from natural sources, such as plants, and their secondary metabolites are efficient quenchers of free radicals and interrupt their production. Their consumption helps in the management of oxidative stress and in preventing the onset of several diseases such as DM and AD [2,36,37,38,39]. Numerous in vitro and in vivo studies have shown that *C. nocturnum* leaf extracts have antifungal, antibacterial, antidiabetic, and wound healing properties [22,24,27,28,40].

In this study, sequential extraction of *C. nocturnum* leaves was performed using *n*-Hexane, DCM, EtOAc, MeOH, and water. Phytochemical screening showed that the MeOH extract contains significant amounts of bioactive compounds, including flavonoids and polyphenols (Table 2), which are known for their free radical quenching ability, and that it has the highest TPC (Figure 2). The reductones serve as antioxidants by donating a hydrogen atom to free radicals, and their content corresponds with the reducing capacity of the extracts and their antioxidant potential [29,30,31]. The MeOH extract exhibited significant total antioxidant activity (Figure 1). These results are in concordance with those of previously studies [22,23,28,40]. A recently published study also showed that leaves of *C. nocturnum* are a rich source of phytochemical constituents [40]. Because phenolic compounds are believed to be responsible for the majority of antioxidant properties of plant extracts, the antioxidant potential of the MeOH extract might be attributable to polyphenolic compounds [32,41]. Compounds with the ability to reduce oxidative stress via quenching of free radicals can delay or stop the progression of several chronic diseases [1,8,42,43]. The MeOH extract of *C. nocturnum* leaves exhibited strong DPPH and ABTS radical quenching ability (Figure 1, Table 3), indicating its potent antioxidant activity. These results are in agreement with previously published reports [23,44,45].

Several strategies have been developed to manage DM, among which, the strategies based on the inhibition of key enzymes are the most common. The inhibition of the most important carbohydrate metabolizing enzymes (α-amylase and α-glucosidase) is the first line drug therapy for the management of blood glucose levels in DM patients [29,46,47]. Oxidative stress and DM contribute to the development of several complications, including cognitive disorders, such as AD. AD is the most common cause of dementia. Epidemiological studies suggest that DM patients are more prone to develop AD [9,48]. The most prominent therapeutic strategy for AD is the inhibition of cholinesterase, as this enzyme catalyzes the conversion of ACh into choline and acetate. Several studies have established a strong relationship between DM, particularly type 2 DM, and AD, as they share common pathophysiological features, such as oxidative stress, abnormal signaling events related to insulin, advanced glycation end products, and mitochondrial anomalies [49,50]. Although the initial management of hyperglycemia is performed through diet control and exercise, this is not sufficient, and oral drug therapy is recommended [51]. Several synthetic drugs are commercially available for the management of hyperglycemia (glinides, carbohydrate metabolizing enzyme inhibitors, sulfonylureas, and thiazolidinediones) [52] and AD (tacrine, donepezil, rivastigmine, and galantamine) [16]. Several studies have shown that these antihyperglycemic drugs reduce the risk of dementia [53,54]. Despite their excellent profile, the long-term use of these anti-diabetes and anti-Alzheimer’s medications causes several prominent side effects, including hepatotoxicity, nephrotoxicity, and hypoglycemia [55,56]. To date, there is no FDA approved drug that can manage both hyperglycemia and AD via targeting of α-amylase and AChE. In this context, our findings that the sequentially extracted *C. nocturnum* extracts exhibit antidiabetic and anti-Alzheimer’s activity via targeting α-amylase and AChE are significant. Among all the extracts, the MeOH extract exhibited the most potent α-amylase inhibitory action. This extract inhibited the α-amylase activity in a dose-dependent manner (Figure 3, Table 3), consistent with previous reports that α-amylase inhibitory potential was higher in more polar plant extracts [32,57,58]. Thus, the enzyme inhibitory potential of the MeOH extract could be due to the presence of polyphenols, flavonoids, and glycosides. Interestingly, the DCM extract also showed marked inhibition at a concentration of 50 µg/mL. Besides this further increasing, the concentration did not show significant inhibitory potential. It might be due to lower number of phytoconstituents in the DCM extract. Preliminary screening also revealed that the MeOH extract of leaves of *C. nocturnum* inhibited the AChE activity in a dose-dependent manner (Figure 3, Table 3).

To find the mechanism of inhibition of α-amylase and AChE by the MeOH extract, we performed enzyme kinetics studies. The MeOH extract was found to be a noncompetitive inhibitor of α-amylase and a competitive inhibitor of AChE (Figure 4). On the contrary, the standard drugs, acarbose and tacrine, showed competitive and noncompetitive inhibition of α-amylase and AChE, respectively, which is in agreement with previous reports [32,39]. It is evident that plant extracts exhibit competitive and noncompetitive inhibition due to the presence of a variety of bioactive compounds [59]. A decrease in V_max_ and no change in K_m_ are characteristics that differentiate noncompetitive inhibition from competitive (no change in V_max_ and an increase in K_m_) and uncompetitive (decrease in both V_max_ and K_m_) inhibition [60].

Using GC-MS analysis, 23 compounds were identified as the bioactive substances probably responsible for the aforementioned effects of the MeOH extract (Table 4). Several studies have reported the antioxidant, antidiabetic, antifungal, and antibacterial activities of these compounds present in *C. nocturnum* [23,40]. However, our GC-MS analysis did not record the flavonol glycoside and steroidal saponins described in an NMR analysis of methanolic extract of leaves by Mimaki et al., (2001) [61]. However, these chemicals were also not documented in a previously published publication either, although our data are consistent with the same class of substances reported by Chaskar et. al. (2017), such as hexadecenoic acid, 1-Hexadecanol, and carboxylic acid [62].

Based on our results, we surmise that the bioactive compounds in the MeOH extract of *C. nocturnum* leaves, either individually or in combination, substantially ameliorate the oxidative damage and inhibit the activities of α-amylase and AChE. However, the most persuasive step in the development of drugs is the prediction of the pharmacological properties of a chemical entity using several AI-based software. Among the various AI-based strategies, ADMET is currently being used to avoid wastage of time, resources, and manpower [61,62]. For this reason, we performed the ADMET analysis to investigate the drug-likeness properties of the bioactive components of the MeOH extract predicted using GC-MS. The SWISS ADME generates results in the form of a BOILED-Egg graph. The white region denotes high gastrointestinal tract absorption of the compounds and the yellow region (yolk) indicates high BBB penetration. The ADMET analysis revealed that all the compounds had acceptable drug-likeness properties and conformed to Lipinski’s rule of five [29]. However, some compounds violated either one or two of these rules, but these violations do not warrant exclusion of these compounds as potential candidates. Only 13 compounds were localized in the BOILED-Egg graph, and these were subjected to toxicity analysis (Table 5). All the compounds were in the range of classified LD_50_ values. Four compounds (dibutyl phthalate, phthalic acid di-isobutyl ester, *p*-chloromethoxybenzene, and precocene I) were active against the carcinogenicity parameter. Precocene I was also active against the immunogenicity parameter. These compounds were eliminated at this level from further docking analysis.

Molecular docking analysis was performed to determine the interactions of the selected constituents of the MeOH extract that interact with the active site of α-amylase and AChE, and consequently inhibit their activity. Such docking analyses to search for molecular targets of constituents in plant extracts have been reported previously [32]. Molecular docking is a crucial tool for examining the interaction of ligands with a target protein and helps in comprehending the mechanisms underlying their binding and inhibitory activities. Redocking co-crystallized acarbose and tacrine into their respective binding sites in α-amylase and AChE allowed us to validate the docking approach (Figure 5 and Figure 6). We found that all the redocked structures interacted with the same amino acids as in the respective crystal structure. The molecular docking study was carried out using Pyrex and further validated using Autodock 4.2. Furthermore, our results illustrated that the selected ten compounds were strongly occupied the active pocket of the α-amylase crystal structure with binding energy (ΔG) values ranging from −3.10 to −6.23 kcal/mol, which is quite a bit better than the standard (ΔG -2.71 kcal/mol) as well as their substrate (ΔG −2.79 kcal/mol). Among these compounds, 7,9-di-tert-butyl-1-oxaspiro [4,5] deca-6,9-diene-2,8-dione was most potent inhibitor of α-amylase, as evidenced by its lowest binding energy. Its binding to the active pocket was stabilized by interaction with nine amino acid residues (Leu165, Gln63, Thr163, Trp58, Trp59, Asp300, His299, Arg195, Tyr62) (Figure 5). Interestingly, the same compound also showed the lowest binding affinity for AChE, and its binding was stabilized through interactions with 17 amino acid residues (Ser122, Asp72, Asn85, Trp84, Ser81, Gly80, Phe330, Tyr334, Tyr442, Trp432, Ile439, Ser200, His440, Glu199, Ile444, Gly441, Tyr121) (Figure 6). The other selected compounds also interacted efficiently with the active pocket of AChE, showing varied ΔG values (Table 8). Although all the selected compounds interacted with the catalytic site of the both the target enzymes, resulting in inhibition of their activity, we cannot comment if all or few of these compounds are responsible for the actual inhibitory activity of the extract. Nonetheless, the results of our in vitro and in silico studies convincingly highlight the antidiabetic and anti-Alzheimer potential of the MeOH extract of *C. nocturnum* leaves.

## 4. Material and Methods

### 4.1. Chemicals

*n*-Hexane, DCM, EtOAc, MeOH, acetone, and dinitro salicylic acid (DNS) were obtained from Merck. DPPH, 2,4,6-tripyridyl-s-triazine (TPTZ), ascorbic acid, ferric chloride (FeCl_3_), and ferrous sulfate (FeSO_4_) were purchased from the Hi-Media Laboratories. Pancreatic α-amylase was obtained from Sisco Research. Lab Pvt. Ltd. DTNB, acetylcholine iodide (AChI), 9-amino-1,2,3,4-tetrahydroacridine hydrochloride (tacrine hydrochloride), ABTS, and AChE were purchased from Sigma-Aldrich (USA). All the chemicals were of analytical grade.

### 4.2. Collection, Identification, and Preparation of Cestrum nocturnum Extract

The *C. nocturnum* leaves were collected (voucher no. IU/PHAR/HRB/22/21) and washed to remove filth and dust particles and shed dried for seven days. After drying, leaves were ground to powder form. The dried powder (25 g) was sequentially extracted with the appropriate amount of *n*-Hexane, dichloromethane (DCM), ethyl acetate (EtOAc), methanol (MeOH), and water using the Soxhlet apparatus. The filtered crude extract was scratched out and kept at −20 °C for further analytical use. The following formula was used to determine the percentage yield of various extracts.

$$\%\;\mathrm{yield}\;=\frac{\mathrm{Weight}\;\mathrm{of}\;\mathrm{crude}\;\mathrm{extract}}{\mathrm{Weight}\;\mathrm{of}\;\mathrm{raw}\;\mathrm{material}}\times 100$$

### 4.3. Qualitative Screening of Phytochemicals

Each extract of leaves of *C. nocturnum* was qualitatively screened for the presence of phytoconstituents, such as phenols, glycosides, and steroids, following the methods described previously [63].

### 4.4. DPPH Radical Scavenging Activity

The method described by Brand-Williams et al. [64] was used to assess the DPPH radical quenching ability of the extracts. The reference standard ascorbic acid was used for the comparative study. The percent inhibition of the DPPH was calculated using the equation below:

$$\%\mathrm{DPPH}=\frac{\Delta \mathrm{Absorbance}\;\mathrm{of}\;\mathrm{control}-\Delta \mathrm{Absorbance}\;\mathrm{of}\;\mathrm{sample}}{\Delta \mathrm{Absorbance}\;\mathrm{of}\;\mathrm{control}}\times 100$$

### 4.5. ABTS Radical Scavenging Activity

The ABTS stock solution (7 mM) was prepared by mixing it with 2.45 mM potassium persulfate. Before the experiment, the solution was suitably diluted to yield an absorbance of 0.70 at 734 nm. Different concentrations of the extracts (in a 100 μL volume) were added to 900 μL of ABTS solution and the mixtures were incubated for 30 min at 37 °C. The absorbance was taken at 734 nm using an Eppendorf Bio-spectrophotometer. The reference standard used was ascorbic acid [65]. The equation used for calculating the % inhibition was the same as that used for DPPH.

### 4.6. Ferric Reducing Antioxidant Potential

The ferric reducing potential was determined according to the standard protocol [66] with a slight modification [32]. The absorbance was taken at 593 nm. The results were calculated using the standard curve of FeSO_4_ and indicated as μmol Fe (II)/g dry weight of the *C. nocturnum* leaves powder.

### 4.7. Total Phenolic Content

The total phenolic content was determined by using the Follin–Ciocalteu standard protocol [32]. The results were calculated using standard gallic acid curve. The results are manifested as μg GA equivalent/mg extract.

### 4.8. α-Amylase Inhibition Assay

The α-amylase inhibitory potential of the different *C. nocturnum* leaf extracts was determined according to the standard protocol [29,32]. The enzyme (5 unit/mL) was freshly prepared in 20 mM of ice-cold PBS (pH 6.7) containing 6.7 mM NaCl. The enzyme (250 μL) was mixed with different concentrations of the inhibitors (acarbose or extract), except in the blank, and incubated for 20 min at 37 °C. Thereafter, starch solution (0.5% *w*/*v*) was added and the mixture was incubated for 15 min at 37 °C. Following the addition of the DNS reagent, the mixture was vortexed and incubated at 100 °C for 10 min in a water bath. At the end of incubation, the absorbance at 540 nm was measured using an Eppendorf Bio-spectrophotometer. The % inhibition rate was evaluated using the following equation:

% inhibition = 100 − % reaction

where % reaction = (mean product in sample/mean product in control) × 100

### 4.9. Determination of Anti-Acetylcholinesterase Activity

The acetylcholinesterase test was prepared according to Ellman et al. (1961) with a slight modification [67]. For use as a blank control, 33 μL of 10 mM DTNB, 100 μL of 1 mM AChI, 767 μL of 50 mM Tris HCl buffer (pH 8.0), and 100 μL of extract (different concentrations) were mixed in a 2 mL cuvette. For the test reaction, 300 μL of the buffer was replaced with an equal volume of AChE solution (0.28 U/mL). Tacrine was used as a reference standard. The reaction was monitored for 20 min by measuring the OD at 405 nm every minute. The values are presented as the mean of three replicates. The % inhibition of enzyme activity was calculated using the following equation:

$$\%\;\mathrm{inhibition}=\frac{\Delta \mathrm{Absorbance}\;\mathrm{of}\;\mathrm{control}-\Delta \mathrm{Absorbance}\;\mathrm{of}\;\mathrm{sample}}{\Delta \mathrm{Absorbance}\;\mathrm{of}\;\mathrm{control}}\times 100$$

### 4.10. Kinetics Studies to Assess the Mode of Inhibition of α-Amylase Activity by the MeOH Extract of C. nocturnum Leaves

Michaelis–Menten kinetics (the Lineweaver–Burk plot) [30,32] were determined to decipher the mode of inhibition of α-amylase activity by the MeOH extract of *C. nocturnum* leaves. α-Amylase was preincubated with the inhibitor (extract/acarbose) for 20 min. One hundred microliters of starch (0.625–5 mg/mL) was added to each tube, including the blank, and incubated at 37 °C for 15 min. After the addition of DNS solution, the absorbance was recorded at 540 nm. The Lineweaver–Burk plot was made to determine the effect of the extract or acarbose on V_max_ and K_m_.

### 4.11. Kinetics Studies to Assess the Mode of Inhibition of AChE Activity by the MeOH Extract of C. nocturnum Leaves

The kinetic study was carried out using the varied concentration of substrate, AchI (i.e., 0.5, 1.0, and 2.0 mM), and inhibitor *C. nocturnum* leaves extract (0.0, 50, and 100 µg/mL of reaction). The hydrolysis of AChI by AChE, either in the absence or presence of an inhibitor, was spectrophotometrically monitored for 20 min at 405 nm. The absorbance was taken at 1 min intervals. The mode of inhibition was determined according to the Michaelis–Menten kinetics [39].

### 4.12. GC-MS Analysis of the MeOH Extract

The phytoconstituents in the MeOH extract, which exhibited the maximum inhibitory potential against α-amylase and AChE, were identified using GC-MS. The GC-MS analysis was performed at the Central Instrumentation Laboratory Facility (CIL), Central University of Punjab, Bhatinda, India. The sample was injected into a Restek column (30 m × 0.25 mm; film thickness, 0.25 μm) on a GC-MS system (Shimadzu QP 2010 Ultra GC-MS). The constant column flow of the carrier gas (helium) was 1 mL/min. The mass spectra peaks were compared against the reference National Institute of Standards and Technology (NIST) libraries to identify the compounds.

### 4.13. Retrieval and Preparation of Ligands Structure

Numerous organic compounds’ structures and their functional details are available in the PubChem database (http://pubchem.ncbi.nlm.nih.gov) that accessed on 10 November 2022. A unique identification number (CID) has been designated for each compound in the database. The 3D-structures of GC-MS-identified compounds were retrieved in 3D SDF file format. Using BIOVIA Discovery Studio Visualizer, the SDF file of ligands was converted into PDB file format. The CHARMM force field was applied in order to singe step minimization using the steepest descent method for 500 steps and an RMS gradient of 0.01.

### 4.14. Preparation of Target Protein

The 3D-structure of both enzymes (target proteins) was downloaded from the PDB database (https://www.rcsb.org/search) that accessed on 10 November 2022 [68] by taking the proteins IDs, α-amylase (5U3A), AChE (1ACJ), and saved. The structure was investigated and visualized in BIOVIA Discovery Studio Visualizer 2020 (BIOVIA, Dassault Systems; https://discover.3ds.com/discovery-studio-visualizer-download, accessed on 12 October 2022. Moreover, an online tool, Play-Molecule (https://www.playmolecule.com) accessed on 8 November 2022, provided the DEEPSITE to predict the active site of the AchE.

### 4.15. Target Protein and Ligands Preparation

The target protein was prepared by deleting heteroatoms and adding polar hydrogen, as well as kollman charges, by using Autodock 4.2 [69]. Further 3D structure of the proteins was converted into PDBQT file format. The ligands were prepared according to the well-defined standard protocol [70].

### 4.16. ADME and Drug-Likeness Studies of Selected Ligands

The selected ligands were subjected to pharmacokinetic profiling by using a web-based tool, as defined in earlier studies [71]. Furthermore, the ligands’ drug-likeness properties were also depicted by the Swiss ADME tool (http://www.swissadme.ch) that has been accessed on 20 November 2022.

### 4.17. Predicted Toxicity of the Selected Compounds

Toxicity prediction was performed by the ProTox-II (https://tox-new.charite.de/protox_II/index.php?site=compound_input) on 25 November 2022, an online web-based server for the prediction of toxicities of small molecules. It provides the numerous details of the compounds about the toxicity such as LD_50_, Carcinogenicity, Immunotoxicity, Mutagenicity Cytotoxicity, as well as, most importantly, Hepatotoxicity [72].

### 4.18. Molecular Interaction Analysis

To determine the antidiabetic and anti-Alzheimer’s potential of the selected compounds, we performed in silico molecular docking of these compounds at the catalytic sites of α-amylase and AChE, respectively, using the standard protocol [73]. For validating the results of docking, the structures of acarbose and tacrine were extracted from the structures of their respective complexes with α-amylase and AChE and redocked within the active pocket of the respective targets using Autodock. After the completion of docking, the structures of the complexes were visualized using the BIOVIA Discovery Studio Visualizer and ranked on the basis of binding energies.

## 5. Conclusions

For the first time, we demonstrate the potent antidiabetic and anti-Alzheimer’s activities of sequentially extracted *C. nocturnum* methanolic leaf extracts via the inhibition of α-amylase and AChE, respectively. The results of our in vitro analyses show that the methanolic extract of *C. nocturnum* leaves has potent antioxidant, antidiabetic, and anti-Alzheimer’s activities. These results were further corroborated by the antidiabetic and anti-Alzheimer’s properties of the bioactive compounds identified using GC-MS. The findings suggest that 7,9-di-tert-butyl-1-oxaspiro [4,5] deca-6,9-diene-2,8-dione alone or in combination with other compounds inhibits the activities of both α-amylase and AChE. Thus, it is a good approach to alleviate oxidative stress and hyperglycemia, as well as Alzheimer’s, with the whole of these compounds/extracts. A thorough and comprehensive in vivo study is also required to fully understand the function of these extracts and their bioactive constituents.

## Figures and Tables

**Table 1:** %Yield of phytochemicals in different extracts of the leaves of *C. nocturnum*.

Extract	%Yield Leaf Extract
*n*-Hexane	1.44
Dichloromethane	1.9
Ethyl acetate	0.71
Methanol	7.63
Aqueous	6.38

**Table 2:** Phytochemical profiling of different *C. nocturnum* leaf extracts.

Phytochemicals	*n*-Hexane	EtOAc	DCM	MeOH	Aqueous
Cardiac glycosides	−	+++	−	+	−
Steroids	−	−	++	−	−
Phenols	++	+++	+	++	−
Flavonoids	+	−	+	+++	+
Tannins	−	+++	−	++	−
Saponins	−	−	−	+++	++
Terpenoids	−	−	+	−	−
Quinone	++	+++	−	+++	−
Coumarins	+++	−	+	+	+
Phlobatannins	−	−	−	−	−
Anthocyanin	−	−	−	+	−

**Table 3:** IC_50_ values of the extracts of *C. nocturnum* leaf against DPPH, ABTS, α-amylase, and AChE inhibition.

Activity	Plant Extract/Standard	IC_50_ (µg/mL)
DPPH	*n*-Hexane	185.67 ± 0.81
	DCM	NS
	EtOAc	NS
	MeOH	39.12 ± 0.53
	Aq.	NS
	Ascorbic acid	15.12 ± 0.65
ABTS	*n*-Hexane	79.13 ± 0.51
	DCM	NS
	EtOAc	50.41 ± 0.76
	MeOH	20.94 ± 0.82
	Aq.	56.73 ± 0.56
	Ascorbic acid	22.76 ± 0.43
α-amylase inhibition	*n*-Hexane	NS
	DCM	NS
	EtOAc	NS
	MeOH	188.77 ± 1.67
	Aq.	NS
	Acarbose	41.54 ± 0.54
Acetylcholinesterase inhibition	*n*-Hexane	NS
	DCM	NS
	EtOAc	NS
	MeOH	239.44 ± 0.93
	Aq.	NS
	Tacrine	4.03 ± 0.47

**Table 4:** GC-MS predicted compounds with their molecular weight, formula, peak area (%), and respective PubChem IDs.

S. No	R.T ^1^	Compound	PubChem ID	Molecular Formula	Molecular Weight	Area %
1	20.058	1-Hexadecanol	2682	C_16_H_34_O	242	2.31
2	20.238	p-Chloromethoxybenzene	12,167	C_7_H_7_ClO	142	1.53
3	21.753	Hexaglycerine	6510	C_6_H_14_O_3_	134	14.46
4	23.234	2,4-Di-tert-butylphenol	7311	C_14_H_22_O	206	20.05
5	23.817	8-Methylpeptadiecance	292,723	C_18_H_38_	254	0.50
6	25.408	Precocene I	28,619	C_12_H_14O2_	190	18.74
7	29.310	DL-Arabinose	854	C_5_H_10_O_5_	150	4.11
8	30.011	Behenic alcohol	12,620	C_22_H_46_O	326	1.87
9	31.471	Phthalic acid, diisobutyl ester	6782	C_16_H_22_O_4_	278	1.85
10	32.457	7,9-Di-tert-butyl-1-oxaspiro (4,5) deca-6,9-diene-2,8-dione	545,303	C_17_H_24_O_3_	276	1.56
11	32.835	Hexadecanoic acid, methyl ester	8181	C_17_H_34_O_2_	270	1.63
12	32.940	Methyl 3-(3,5-ditert-butyl-4-hydroxyphenyl) propanoate	62,603	C_18_H_28_O_3_	292	6.73
13	33.482	Dibutyl phthalate	3026	C_16_H_22_O_4_	278	2.44
14	44.487	2-Palmitoylglycerol	123,409	C_19_H_38_O_4_	330	1.18
15	47.464	Docosanoic anhydride	566,696	C_44_H_86_O_3_	662	2.34
16	50.251	Dimethyl Eicosanedioate	566,668	C_22_H_42_O_4_	370	3.70
17	52.870	Tetracontanedioic acid, dimethyl ester	566,763	C_42_H_82_O_4_	650	2.77
18	55.677	Docosanoic acid	8215	C_22_H_44_O_2_	340	1.49
19	55.790	2,4,6-Trichlorobenzoic acid	5764	C_7_H_3_Cl_3_O_2_	224	0.18
20	56.753	Sebacic acid, di(4-bromo-2,6-difluorobenzyl) ester	91,729,085	C_24_H_24_Br_2_F_4_O_4_	610	0.57
21	59.743	Beta-Sitosterol trimethylsilyl ether	14,429,144	C_32_H_58_OSi	486	0.10
22	60.173	Tris(2,4-di-tert-butylphenyl) phosphate	14,572,930	C_42_H_63_O_4_P	662	0.45
23	61.523	Methyl 22-hydroxydocosanoate	13,406,065	C_23_H_46_O_3_	370	0.32

^1^ Retention time.

**Table 5:** Chemical properties of GC-MS predicted compounds of *C. nocturnum*.

S. No.	Compound Name	PubChem ID (CID)	Log P ^1^	TPSA ^2^ (Å^2^)	BBB ^3^	HIA ^4^	HBA ^5^	HBD ^6^	Rotatable Bonds	Violation
1	1-Hexadecanol	2682	4.41	20.23	Yes	High	1	1	14	1
2	p-Chloromethoxybenzene	12,167	2.18	9.23	No	High	1	0	1	1
3	Hexaglycerine	6510	1.26	60.69	No	High	3	3	4	0
4	2,4-Di-tert-butylphenol	7311	3.08	20.23	Yes	High	1	1	2	0
5	8-Methylpeptadicance	292,723	5.13	0	Yes	Low	0	0	14	0
6	Precocene I	28,619	2.74	18.46	Yes	High	2	0	1	0
7	DL-Arabinose	854	0.1	97.99	No	Low	5	4	4	0
8	Behenic alcohol	12,620	5.73	20.23	No	Low	1	1	20	1
9	Phthalic acid, diisobutyl ester	6782	3.31	52.6	Yes	High	4	0	8	0
10	7,9-Di-tert-butyl-1-oxaspiro (4,5) deca-6,9-diene-2,8-dione	545,303	2.91	43.37	Yes	High	3	0	2	0
11	Hexadecanoic acid, methyl ester	8181	4.41	26.3	Yes	High	2	0	15	1
12	Methyl 3-(3,5-ditert-butyl-4-hydroxyphenyl) propanoate	62,603	3.75	46.53	Yes	High	3	1	6	0
13	Dibutyl phthalate	3026	2.97	52.6	Yes	High	4	0	10	0
14	2-Palmitoylglycerol	123,409	4.5	66.76	Yes	High	4	2	18	0
15	Docosanoic anhydride	566,696	10.4	43.37	No	Low	3	0	42	2
16	Dimethyl Eicosanedioate	566,668	5.27	52.6	No	High	4	0	21	1
17	Tetracontanedioic acid, dimethyl ester	566,763	10.06	52.6	No	Low	4	0	41	2
18	Docosanoic acid	8215	5.26	37.3	No	Low	2	1	20	1
19	2,4,6-Trichlorobenzoic acid	5764	1.62	37.3	Yes	High	2	1	1	0
20	Sebacic acid, di(4-bromo-2,6-difluorobenzyl) ester	91,729,085	5.42	52.6	No	Low	8	0	15	2
21	Beta-Sitosterol trimethylsilyl ether	14,429,144	6.36	9.23	No	Low	1	0	8	1
22	Tris(2,4-di-tert-butylphenyl) phosphate	14,572,930	6.93	54.57	No	Low	4	0	12	2
23	Methyl 22-hydroxydocosanoate	13,406,065	5.8	46.53	No	Low	3	1	22	1
24	Tacrine	1935	2.09	38.91	Yes	High	1	1	0	0
25	Acarbose	41,774	1.43	321.17	No	Low	19	14	9	3

^1^ Log P, octanol–water partition coefficient; ^2^ TPSA, topological polar surface area; ^3^ BBB, blood–brain barrier; ^4^ HIA, human intestinal absorption; ^5^ HBA, hydrogen-bond acceptor; ^6^ HBD, hydrogen-bind acceptor.

**Table 6:** Toxicity of the selected compounds.

S. No.	Compound Name	PubChem ID (CID)	LD_50_ (mg/kg)	Toxicity Class	Hepatotoxicity	Carcinogenicity	Immunogenicity	Mutagenicity	Cytotoxicity
1	1-Hexadecanol	2682	1000	4	Inactive	Inactive	Inactive	Inactive	Inactive
2	2,4-Di-tert-butylphenol	7311	700	4	Inactive	Inactive	Inactive	Inactive	Inactive
3	2,4,6-Trichlorobenzoic acid	5764	830	4	Inactive	Inactive	Inactive	Inactive	Inactive
4	2-Palmitoylglycerol	123,409	5000	5	Inactive	Inactive	Inactive	Inactive	Inactive
5	7,9-Di-tert-butyl-1-oxaspiro (4,5) deca-6,9-diene-2,8-dione	545,303	900	4	Inactive	Inactive	Inactive	Inactive	Inactive
6	Hexadecanoic acid, methyl ester	8181	5000	5	Inactive	Inactive	Inactive	Inactive	Inactive
7	Dimethyl Eicosanedioate	566,668	5000	5	Inactive	Inactive	Inactive	Inactive	Inactive
8	Hexaglycerine	6510	12,980	6	Inactive	Inactive	Inactive	Inactive	Inactive
9	Methyl 22-hydroxydocosanoate	13,406,065	5000	5	Inactive	Inactive	Inactive	Inactive	Inactive
10	Dibutyl phthalate	3026	3474	5	Inactive	Active	Inactive	Inactive	Inactive
12	Phthalic acid, diisobutyl ester	6782	10,000	6	Inactive	Active	Inactive	Inactive	Inactive
13	p-Chloromethoxybenzene	12,167	318	4	Inactive	Active	Inactive	Inactive	Inactive
14	Precocene I	28,619	500	4	Inactive	Active	Active	Inactive	Inactive

**Table 7:** Amino acid residues within the active pocket of α-amylase predicted to interact with the selected compounds.

S. No.	Compound Name	CID	Binding Energy	Inhibition Constant	Interacting Amino Acid
1	1- Hexadecanol	2682	−4.47	529.44 µM	Gln63, Gly104, Thr163, Trp59, Lue165, His101, Glu233, Ser199, Val234, Lys200, Ile235, His201, Leu162, Asp197, Ala198, Tyr62.
2	2,4,6-Trichlorobenzoic acid	5764	−4.77	319.09 µM	Asp197, His201, Tyr151, Lys200, Leu162, Val234, Ala198, Glu233, Ile235,
3	Hexaglycerine	6510	−3.18	4.68 mM	Lys178, Ala128, Tyr67, Ser66, Val129, Lys68, Tyr182, Glu181,
4	2,4 di-tert-butylphenol	7311	−5.54	86.69 µM	Tyr62, His299, Trp59, Leu165, Gln63, His101, Leu162, Ala198, Glu233, Asp197, Arg195, Asp300, Trp58,
5	Palmitic acid Methyl ester	8181	−3.60	2.28 mM	Gln63, Trp59, Trp58, Asp356, Arg303, His305, Trp357, Leu165, Asp300, Leu162, His299, Arg195, Asp197, Tyr62
6	2-palmitoylglycerol	123,409	−4.01	1.15 mM	His101, His299, Tyr62, Leu162, Leu165, Gln63, Asp300, Trp59, Trp357, Asp356, Arg303, Trp58, His305, Glu233, Arg195, Ala198, Asp197
7	7,9-Di-tert-butyl-1-oxaspiro [4.5] deca-6,9-diene-2,8-dione	545303	−6.23	27.20 µM	Leu165, Gln63, Thr163, Trp58, Trp59, Asp300, His299, Arg195, Tyr62
8	Dimethyl Eicosanedioate	566,668	−4.13	940.74 µM	Gly104, Thr163, Leu165, Tyr62, Gln63, Trp59, Ala50, Val107, Tyr52, Ala106, Ile51, Asn53, Val59, Ser108
9	Methyl 22-hydroxydocosanoate	13,406,065	−3.10	5.13 mM	Ile51, Asn53, Tyr52, Val107, Glu233, Asp197, Arg195, Ala198, Leu162, His101, Leu165, Tyr62, Trp59, Thr163, Gln63, Gly104
10	Acarbose *	41,774	−2.71	10.28 mM	Asp300, Trp58, Leu165, Gly164, Asn105, Gly104, Ala106, Val107, Ile51, Trp59, Gln63, Thr163, Leu162, Asp197, Ala198, His201. Ile235, Glu233, Asn298, Arg195
11	Starch ^#^	51,003,661	−2.79	9.06 mM	Asn53, Ser108, Tyr52, Val107, Ala50, Ser112, Ala106, Val49, Ile51,Gly104, Gln63, Trp59, Pro54

* Standard inhibitor; ^#^ Standard substrate.

**Table 8:** Amino acid residues within the active pocket of acetylcholinesterase (AChE) predicted to interact with the selected compounds.

S. No.	Compound Name	CID	Binding Energy	Inhibition Constant	Interacting Amino Acid
1	1-Hexadecanol	2682	−5.88	49.30 µM	Ser200, Glu199, His440, Tyr442, Phe330, Ile439, Tyr334, Ser81, Gly80, Trp432, Ile444, Trp84, Gly441, Ser122, Tyr121, Gly118, Tyr130, Gly117
2	2,4,6-Trichlorobenzoic acid	5764	−5.21	152.67 µM	Gly441, Trp432, Ile439, Ser81, Gly80, Tyr442, Tyr334, Asp72, Trp84, His440, Phe330,
3	Hexaglycerine	6510	−3.32	3.66 mM	Ile287, Phe290, Ser286, Arg289, Phe288, Leu282, Trp279, Tyr334, Phe331
4	2,4 di-tert-butylphenol	7311	−7.20	5.24 µM	Gly441, Phe330, His440, Ile439, Met436, Tyr442, Tyr334, Trp432, Ser81, Gly80, Trp84, Gly118
5	Palmitic acid Methyl ester	8181	−6.14	31.79 µM	Gly118, Asn85, Asp72, Tyr70, Tyr181, Ser122, Tyr334, Trp432, Trp84, Glu199, His440, Tyr442, Ser81, Gly80, Ile439, Gly441, Phe330
6	2-palmitoylglycerol	123,409	−5.10	182.70 µM	Tyr121, Asp72, Tyr334, Tyr70, Phe290, Ser286, Ile287, Phe288, Arg289, Phe330, Phe331, Gly123, Trp279, Leu127, Ser124, Gly177, Ser122, Gly118, Trp84,
7	7,9-Di-tert-butyl-1-oxaspiro [4.5] deca-6,9-diene-2,8-dione	545,303	−8.76	376.56 nM	Ser122, Asp72, Asn85, Trp84, Ser81, Gly80, Phe330, Tyr334, Tyr442, Trp432, Ile439, Ser200, His440, Glu199, Ile444, Gly441, Tyr121,
8	Dimethyl Eicosanedioate	566,668	−6.62	14.01 µM	Glu199, Ile444, Gly117, Tyr130, Gly118, Ser200, His440, Trp84, Gly119, Phe330, Tyr334, Tyr121, Asp72, Trp279, Phe290, Ser291, Leu282, Arg289, Phe288, Ile287, Phe331, Tyr70
9	Methyl 22-hydroxydocosanoate	13,406,065	−6.16	30.33 µM	Arg289, Gly335, Ile287, Asp72, Glu199, Ser200, His440, Gly441, Ile439, Trp84, Gly80, Ser81, Tyr442, Phe330, Trp432, Tyr334, Tyr70, Trp279, Tyr121, Phe331, Leu282, Ser286
10	Tacrine *	1935 *	−8.26	887.31 nM	Tyr442, Gly441, Ser200, Glu199, Gly118, Trp84, Asp72, Ser81, Gly80, Tyr334, Trp432, Phe330, Ile439, His440
11	Acetylcholine Iodide ^#^	187	−4.78	314.07 µM	Phe330, Trp432, Met436, Ile439, Tyr442, His440, Trp84, Asp72, Ser81, Asn85, Tyr334

* Standard inhibitor; ^#^ Standard substrate.

## Data Availability

Not Applicable.

## Supplementary Materials

The following supporting information can be downloaded at: https://www.mdpi.com/article/10.3390/plants12061206/s1, File S1: Chromatograms of GC-MS identified compounds (As Listed in Main text of Manuscript).
