# Supplementary material for: Secondary Metabolite Profiling, Antioxidant, Antidiabetic and Neuroprotective Activity of Cestrum nocturnum (Night Scented-Jasmine): Use of In Vitro and In Silico Approach in Determining the Potential Bioactive Compound
Source: Plants (Basel). 2023 Mar 7;12(6):1206. doi: 10.3390/plants12061206 (PMC10051713; doi:10.3390/plants12061206)

S1: Chromatograms of GC-MS identified compounds (As Listed in Main text of Manuscript)

Hit#:1 Entry:27901 Library:NIST17R.lib
SI:93 Formula:C16H34O CAS:36653-82-4 MolWeight:242 RetIndex:1854
CompName:1-Hexadecanol $$ n-Cetyl alcohol $$ n-Hexadecan-1-ol $$ n-Hexadecanol $$ n-1-Hexadecanol $$ Adol 52 $$ Adol 52 NF $$ Adol 54 $$ Aldo

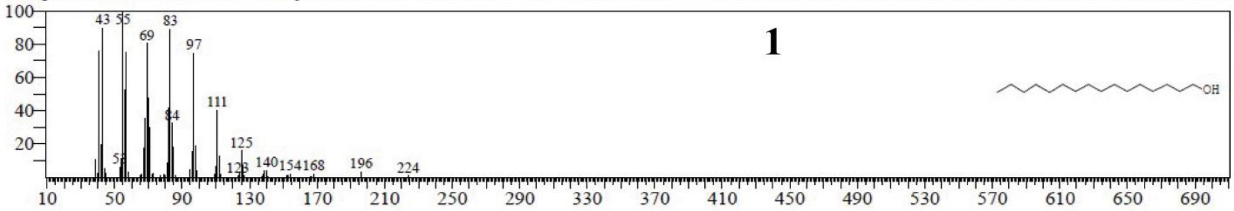

Hit#:1 Entry:9013 Library:NIST17R.lib
SI:65 Formula:C7H7ClO CAS:623-12-1 MolWeight:142 RetIndex:1049
CompName:Benzene, 1-chloro-4-methoxy- $$ Anisole, p-chloro- $$ p-Chloroanisole $$ p-Chloromethoxybenzene $$ p-Chlorophenyl methyl ether $$ Anisy

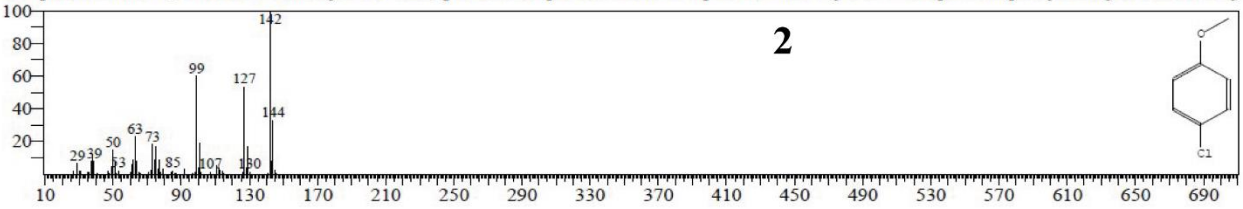

Hit#:1 Entry:7261 Library:NIST17R.lib
SI:75 Formula:C6H14O3 CAS:77-99-6 MolWeight:134 RetIndex:1261
CompName:1,3-Propanediol, 2-ethyl-2-(hydroxymethyl)- $$ Ethriol $$ Ethyltrimethylolmethane $$ Etriol $$ Ettriol $$ Hexaglycerine $$ Propane, 1,1,1-tris

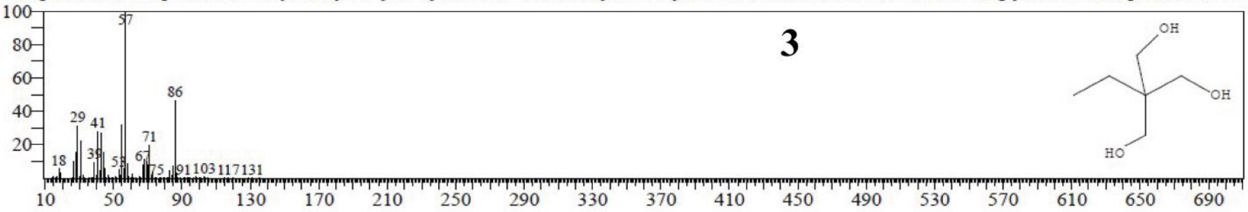

Hit#:1 Entry:22785 Library:NIST17R.lib
SI:96 Formula:C14H22O CAS:96-76-4 MolWeight:206 RetIndex:1555
CompName:2,4-Di-tert-butylphenol $$ Phenol, 2,4-bis(1,1-dimethylethyl)- $$ Phenol, 2,4-di-tert-butyl- $$ 2,4-di-t-Butylphenol $$ 1-Hydroxy-2,4-di-tert-bu

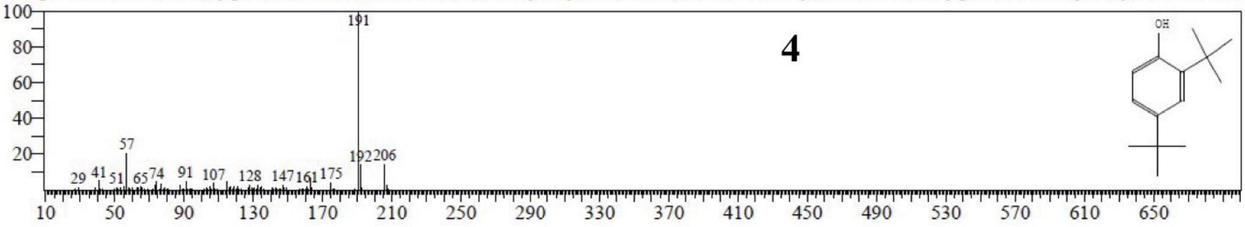

Hit#:1 Entry:29141 Library:NIST17R.lib
SI:66 Formula:C18H38 CAS:13287-23-5 MolWeight:254 RetIndex:1746
CompName:Heptadecane, 8-methyl- $$ 8-Methylheptadecane

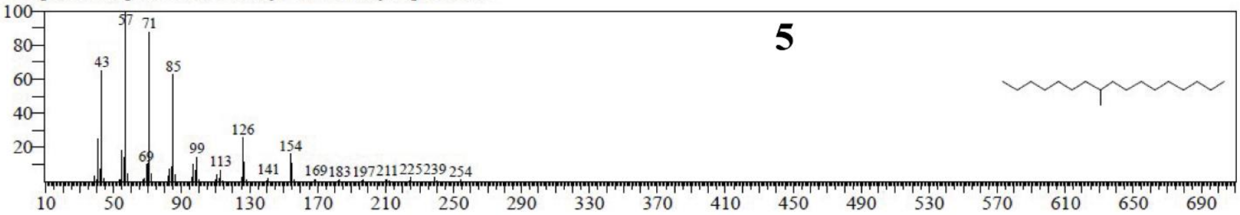

Hit#:1 Entry:19532 Library:NIST17R.lib
SI:70 Formula:C12H14O2 CAS:17598-02-6 MolWeight:190 RetIndex:1461
CompName:Precocene I $$ 2H-1-Benzopyran, 7-methoxy-2,2-dimethyl- $$ Demethoxyageratochromene $$ 6-Demethoxyageratochromene $$ 7-Methoxy-2

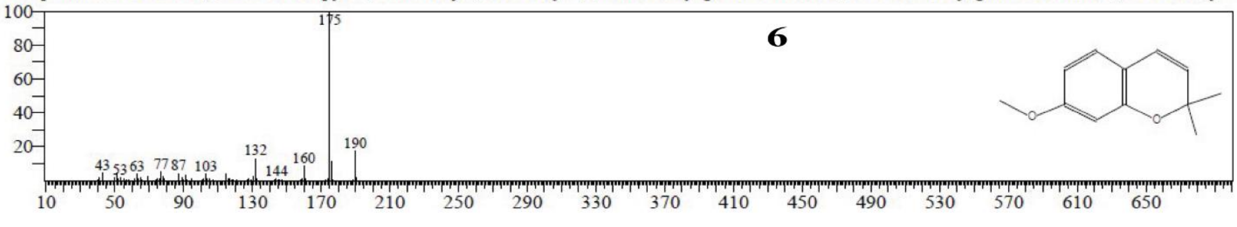

Hit#:1 Entry:10526 Library:NIST17R.lib
SI:75 Formula:C5H10O5 CAS:20235-19-2 MolWeight:150 RetIndex:1461
CompName:DL-Arabinose $$ Pentopyranose #

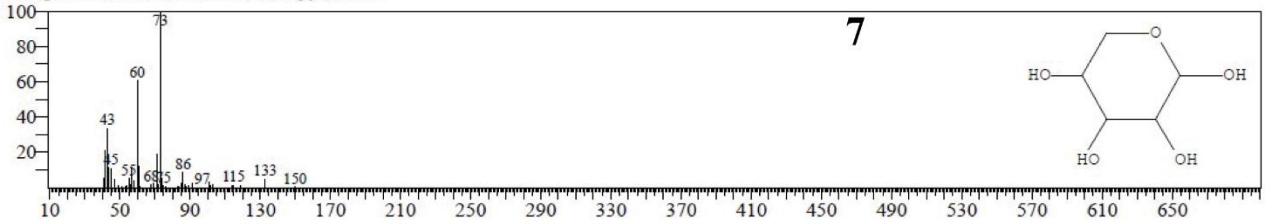

Hit#:1 Entry:34738 Library:NIST17R.lib
SI:91 Formula:C22H46O CAS:661-19-8 MolWeight:326 RetIndex:2451
CompName:Behenic alcohol $$ 1-Docosanol $$ Docosyl alcohol $$ Docosanol-(1) $$ Cachalot BE-22 $$ Loxiol VPG 1451 $$ Docosan-1-ol $$ Behenyl a

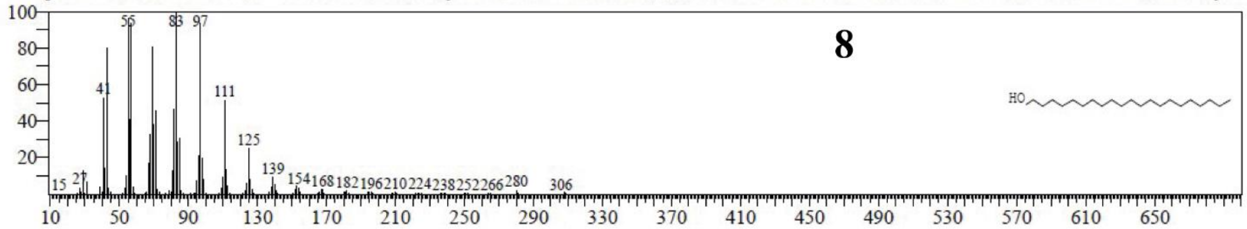

Hit#:1 Entry:31275 Library:NIST17R.lib
SI:89 Formula:C16H22O4 CAS:84-69-5 MolWeight:278 RetIndex:1908
CompName:1,2-Benzenedicarboxylic acid, bis(2-methylpropyl) ester $$ Phthalic acid, diisobutyl ester $$ Diisobutyl phthalate $$ Hexaplas M/1B $$ Isobuty

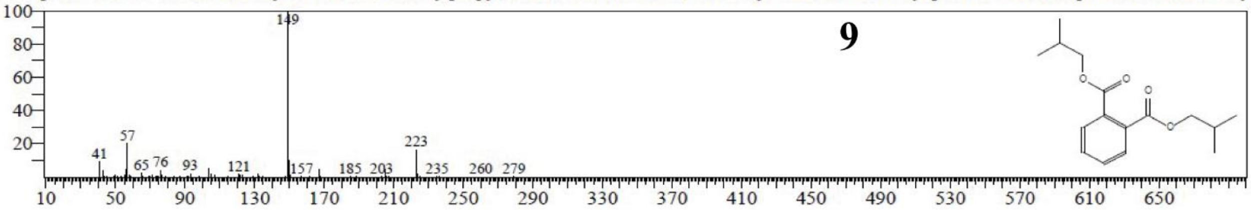

Hit#:1 Entry:31118 Library:NIST17R.lib
SI:85 Formula:C17H24O3 CAS:82304-66-3 MolWeight:276 RetIndex:2081
CompName:7,9-Di-tert-butyl-1-oxaspiro(4,5)deca-6,9-diene-2,8-dione $$ 1-Oxa-spiro[4.5]deca-6,9-diene-2,8-dione, 7,9-di-tert-butyl- $$ 7,9-Di-tert-butyl-1

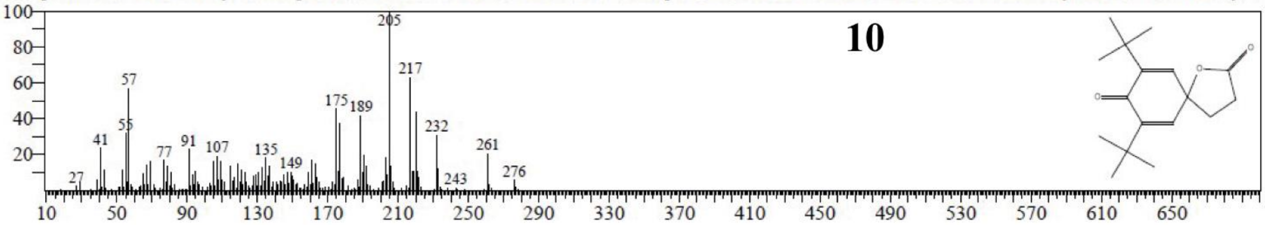

Hit#:1 Entry:30646 Library:NIST17R.lib
SI:81 Formula:C17H34O2 CAS:112-39-0 MolWeight:270 RetIndex:1878
CompName:Hexadecanoic acid, methyl ester $$ Palmitic acid, methyl ester $$ n-Hexadecanoic acid methyl ester $$ Metholene 2216 $$ Methyl hexadecano

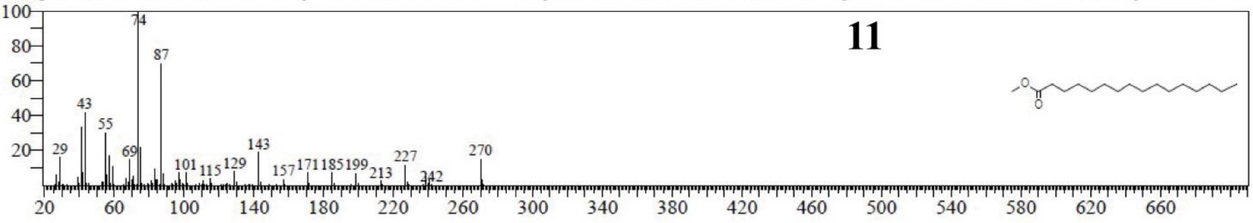

Hit#:1 Entry:32424 Library:NIST17R.lib
SI:90 Formula:C18H28O3 CAS:6386-38-5 MolWeight:292 RetIndex:2134
CompName:Benzenepropanoic acid, 3,5-bis(1,1-dimethylethyl)-4-hydroxy-, methyl ester $$ Methyl 3-(3,5-di-tert-butyl-4-hydroxyphenyl)propionate $$ Met

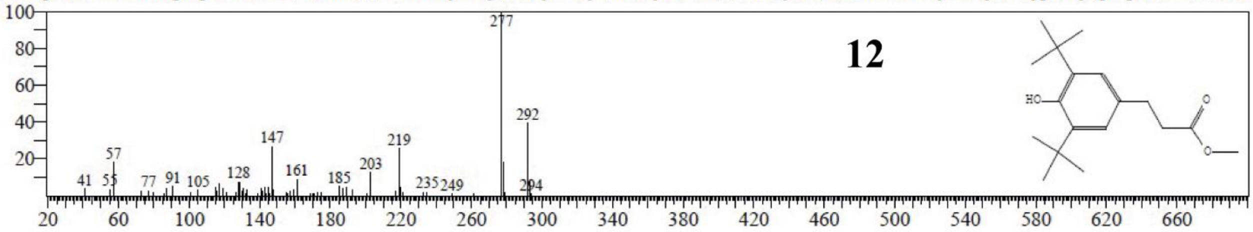

Hit#:1 Entry:31277 Library:NIST17R.lib
SI:94 Formula:$C_{16}H_{22}O_4$ CAS:84-74-2 MolWeight:278 RetIndex:2037
CompName:Dibutyl phthalate $$ 1,2-Benzenedicarboxylic acid, dibutyl ester $$ Phthalic acid, dibutyl ester $$ n-Butyl phthalate $$ Butyl phthalate $$ Cellu

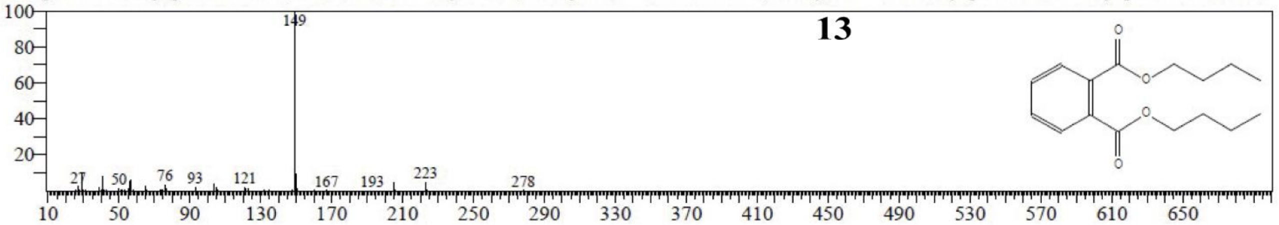

Hit#:1 Entry:34924 Library:NIST17R.lib
SI:56 Formula:$C_{19}H_{38}O_4$ CAS:23470-00-0 MolWeight:330 RetIndex:2498
CompName:Hexadecanoic acid, 2-hydroxy-1-(hydroxymethyl)ethyl ester $$ Palmitin, 2-mono- $$ Palmitic acid .beta.-monoglyceride $$ 2-Hexadecanoyl gl

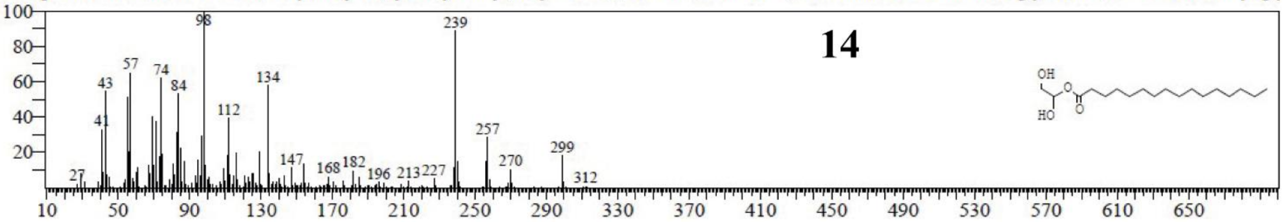

Hit#:1 Entry:3711 Library:NIST17M2.lib
SI:63 Formula:$C_{44}H_{86}O_3$ CAS:55726-23-3 MolWeight:662 RetIndex:4698
CompName:Docosanoic anhydride

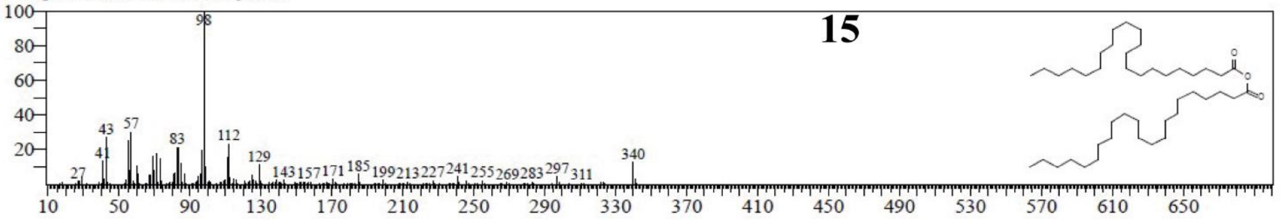

Hit#:1 Entry:36757 Library:NIST17R.lib
SI:61 Formula:$C_{22}H_{42}O_4$ CAS:42235-38-1 MolWeight:370 RetIndex:2543
CompName:Eicosanebioic acid, dimethyl ester $$ Dimethyl icosanedioate #

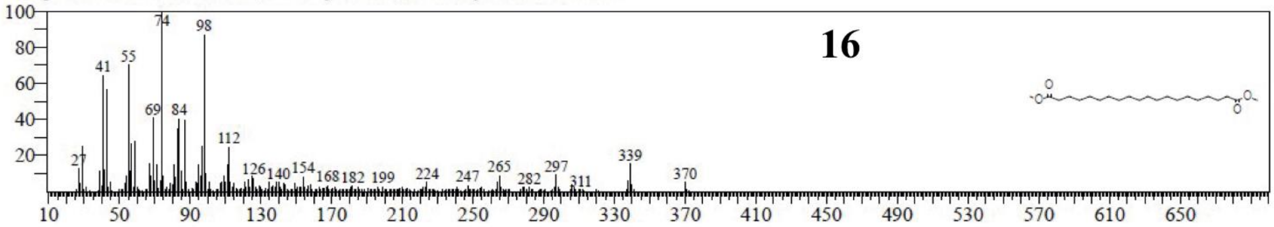

Hit#:1 Entry:3489 Library:NIST17M2.lib
SI:53 Formula:$C_{42}H_{82}O_4$ CAS:0-00-0 MolWeight:650 RetIndex:4531
CompName:Tetracontanedioic acid, dimethyl ester $$ Dimethyl tetracontanedioate #

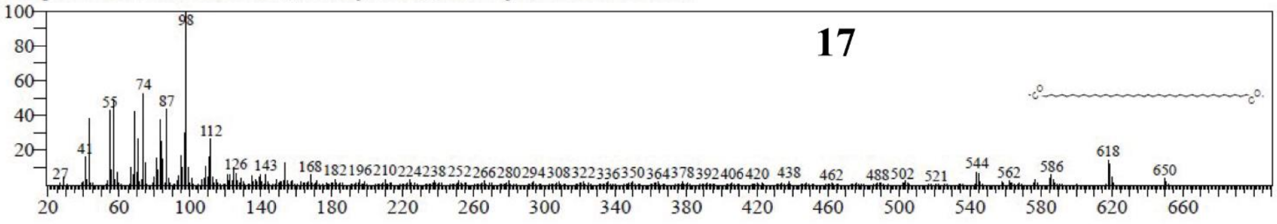

Hit#:1 Entry:35511 Library:NIST17R.lib
SI:46 Formula:$C_{22}H_{44}O_2$ CAS:112-85-6 MolWeight:340 RetIndex:2564
CompName:Docosanoic acid $$ n-Docosanoic acid $$ Behenic acid $$ Glycon B-70 $$ Hydrofol Acid 560 $$ Hydrofol 2022-55 $$ 1-Docosanoic acid $$

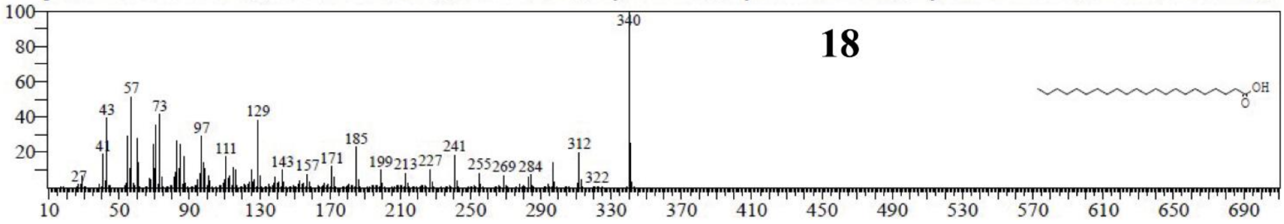

Hit#:1 Entry:25526 Library:NIST17R.lib
SI:39 Formula:C7H3Cl3O2 CAS:50-43-1 MolWeight:224 RetIndex:1690
CompName:Benzoic acid, 2,4,6-trichloro- $$ 2,4,6-Trichlorobenzoic acid

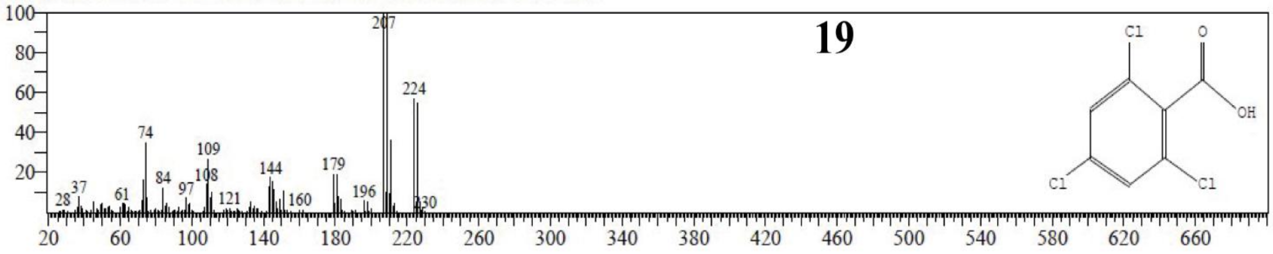

Hit#:1 Entry:2493 Library:NIST17M2.lib
SI:36 Formula:C24H24Br2F4O4 CAS:0-00-0 MolWeight:610 RetIndex:3434
CompName:Sebacic acid, di(4-bromo-2,6-difluorobenzyl) ester

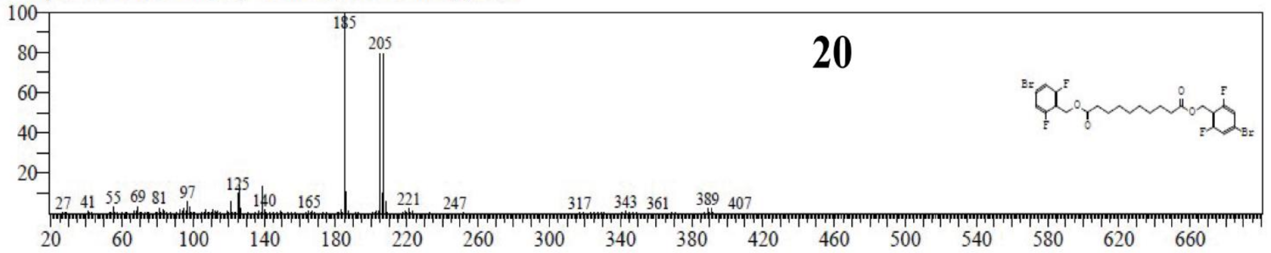

Hit#:1 Entry:38707 Library:NIST17R.lib
SI:38 Formula:C32H58OSi CAS:2625-46-9 MolWeight:486 RetIndex:2789
CompName:.beta.-Sitosterol, TMS derivative $$ .beta.-Sitosterol trimethylsilyl ether $$ Silane, trimethyl[[(3.beta.)-stigmast-5-en-3-yl]oxy]- $$ Silane, trime

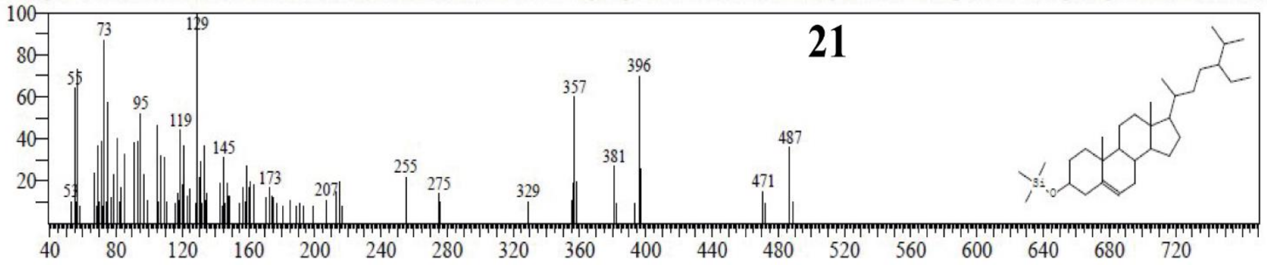

Hit#:1 Entry:3710 Library:NIST17M2.lib
SI:48 Formula:C42H63O4P CAS:95906-11-9 MolWeight:662 RetIndex:0
CompName:Tris(2,4-di-tert-butylphenyl) phosphate $$ Phenol, 2,4-bis(1,1-dimethylethyl)-, 1,1',1''-phosphate $$ Phenol, 2,4-bis(1,1-dimethylethyl)-, phosph

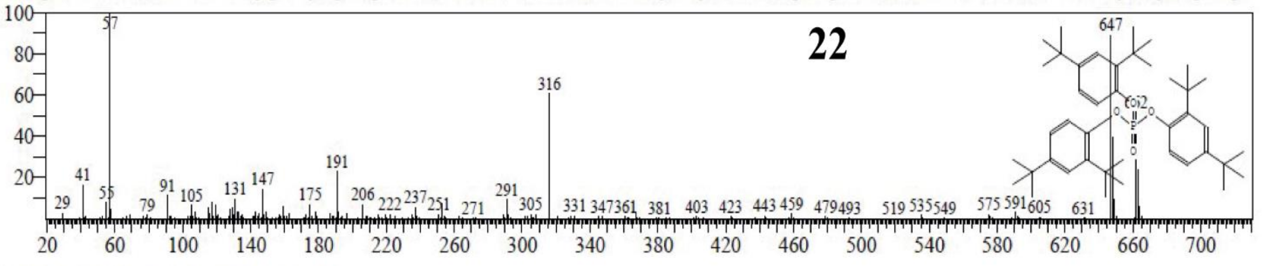

Hit#:1 Entry:36773 Library:NIST17R.lib
SI:38 Formula:C23H46O3 CAS:38646-51-4 MolWeight:370 RetIndex:2717
CompName:Methyl 22-hydroxydocosanoate $$ Methyl .omega.-hydroxydocosanoate $$ Docosanoic acid, 22-hydroxy-, methyl ester

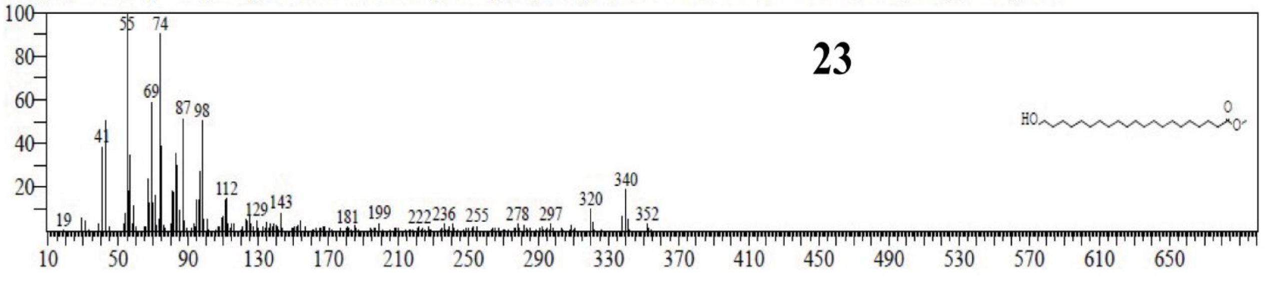

Supplement: Supplementary file 1 [file plants-12-01206-s001.zip › plants-2171766-supplementary.pdf]
